# Neurostereology protocol for unbiased quantification of neuronal injury and neurodegeneration

**DOI:** 10.3389/fnagi.2015.00196

**Published:** 2015-10-31

**Authors:** Victoria M. Golub, Jonathan Brewer, Xin Wu, Ramkumar Kuruba, Jenessa Short, Maunica Manchi, Megan Swonke, Iyan Younus, Doodipala Samba Reddy

**Affiliations:** Department of Neuroscience and Experimental Therapeutics, Texas A&M University Health Science Center, College of MedicineBryan, TX, USA

**Keywords:** stereology, optical fractionator, optical disector, cavalieri estimator, neuronal injury

## Abstract

Neuronal injury and neurodegeneration are the hallmark pathologies in a variety of neurological conditions such as epilepsy, stroke, traumatic brain injury, Parkinson’s disease and Alzheimer’s disease. Quantification of absolute neuron and interneuron counts in various brain regions is essential to understand the impact of neurological insults or neurodegenerative disease progression in animal models. However, conventional qualitative scoring-based protocols are superficial and less reliable for use in studies of neuroprotection evaluations. Here, we describe an optimized stereology protocol for quantification of neuronal injury and neurodegeneration by unbiased counting of neurons and interneurons. Every 20th section in each series of 20 sections was processed for NeuN(+) total neuron and parvalbumin(+) interneuron immunostaining. The sections that contain the hippocampus were then delineated into five reliably predefined subregions. Each region was separately analyzed with a microscope driven by the stereology software. Regional tissue volume was determined by using the Cavalieri estimator, as well as cell density and cell number were determined by using the optical disector and optical fractionator. This protocol yielded an estimate of 1.5 million total neurons and 0.05 million PV(+) interneurons within the rat hippocampus. The protocol has greater predictive power for absolute counts as it is based on 3D features rather than 2D images. The total neuron counts were consistent with literature values from sophisticated systems, which are more expensive than our stereology system. This unbiased stereology protocol allows for sensitive, medium-throughput counting of total neurons in any brain region, and thus provides a quantitative tool for studies of neuronal injury and neurodegeneration in a variety of acute brain injury and chronic neurological models.

## Introduction

Neuronal injury and neurodegeneration are hallmark pathologies of a variety of acute and chronic neurological conditions such as traumatic brain injury, stroke and epilepsy. Neuronal injury or cell death refers to many acute and chronic neurological conditions, including Alzheimer’s disease (AD), Parkinson’s disease (PD) and Huntington’s disease (HD), stroke, trauma, epilepsy, multiple sclerosis and amyotrophic lateral sclerosis (ALS). Acute neurotoxicity, brain ischemia and trauma induce necrosis of a small brain area, which then propagates neuronal cell loss by apoptosis to a larger brain area. Apoptotic death of hippocampal and cortical neurons is responsible for the symptoms of AD; death of midbrain neurons that use the neurotransmitter dopamine underlies PD; HD involves the death of neurons in the striatum, which control body movements; and death of lower motor neurons manifests as ALS. Neurodegeneration refers to the progressive loss of nerve cells, occurring in aging and in neurodegenerative disorders, comprising AD, PD, ALS, HD, stroke, and head trauma. These diseases are characterized by chronic and progressive loss of neurons in discrete areas of the brain, causing debilitating symptoms.

Quantification of absolute neuron and interneuron counts in various brain regions using an optimized neurostereology protocol is essential to understanding the impact of neurological insults or disease progression on neuronal survival and neurodegeneration (West et al., 1991). The conventional qualitative scoring-based protocols are superficial and less reliable for use in studies of neuroprotection evaluations. Over the years, advances in neurostereology methods have been developed to allow for unbiased total estimates of cell numbers in a specific brain region (Keuker et al., 2001). These methods depend on the combination of the optical disector and fractionator sampling scheme. Before the introduction of the disector (Sterio, 1984), neuronal nuclei counting was based on isolated two-dimensional (2D) probes, as neuronal cells only contain one nuclei. Several such two-dimensional probe methods were described previously (Floderus, 1944; Abercrombie, 1946; Weibel, 1969; Rose and Rohrlich, 1987; West et al., 1991). However, the number of nuclei in the brain is a zero-dimensional quantity, hence the various 2D probe methods led to bias. Those concerns were solved by the implementation of the optical disector, a 3D probe (Sterio, 1984; Harding et al., 1993).

Stereology technique allows for reliable quantitative description of a 3D object to be made from 2D measurements. This is achieved by creating a *Z*-stack of 2D images in order to create a 3D tissue model (West et al., 1991; West, 2012a). Stereology utilizes random, systematic sampling to provide unbiased and quantitative data. Modern design-based stereology has been an important and efficient tool in many applications of optical and confocal microscopy. There is a multitude of uses for stereology in many biomedical fields including histology, bone and neuroanatomy to accurately quantify the number of cells, the length of fibers, and the area and volume of biological structures or regions (Mayhew, 2014; Bronoosh et al., 2015).

Sampling is one of the most important statistics-based concepts in stereology (Cruz-Orive and Weibel, 1981; West, 2012b). The application of sampling in stereology is found in every step of the process—from Cavalieri’s estimation of volume to the optical fractionator. The Cavalieri estimator is commonly used to estimate volume of a specific region of the brain such as the hippocampus; however; it can be used to find the volume of any irregular shape of tissue. The estimator is frequently used in conjunction with the optical disector and optical fractionator to estimate densities such as cell number density, length density, or surface density. The optical disector uses a reference volume generated by two parallel sections separated by a known distance to count cells in a 3D space under the microscope (Sterio, 1984; Cruz-Orive, 2004; Kaplan et al., 2012). The counts are considered unbiased as they are unaffected by variations such as size, shape, and orientation of the particles being counted (Sterio, 1984; West et al., 1991; Harding et al., 1993). Subsequently, as the sample size increases, the unbiased cell number estimate for a given reference volume more closely approaches the true value (Kaplan et al., 2012).

Neuronal numbers are estimated by using the combination of the optical disector with the fractionator sampling scheme. The fractionation process involves the counting of neuronal nuclei with an optical disector in a strictly random and systematic sampling scheme that covers a known fraction of the region being analyzed (Cruz-Orive, 1997). The fractionator’s calculations do not require the exact thickness or the exact area of the section being analyzed; therefore, fractionator sampling provides unbiased neuronal counts independent of shrinkage, expansion, and dimensional changes in the tissue (Braendgaard and Gundersen, 1986; West et al., 1991). Systematic sampling consists of sampling the hippocampal region at a random starting position, utilizing a defined periodicity in which all portions in the region have an equal probability of being sampled (Gundersen et al., 1999). The systematic sampling scheme requires far less sampling for an accurate estimate of nuclei in an individual animal. Neuronal counts are estimated using tissue volume, cell density as quantified by the Cavalieri estimator, optical disector, optical fractionator, and sampling scheme (West, 2012a, 2013; Geuna and Herrera-Rincon, 2015).

In this study, we describe an optimized neurostereology protocol for quantification of neuronal injury and neurodegeneration by absolute quantification of principle neurons and interneurons in the brain. The protocol not only implements the optical disector and the fractionator sampling techniques, but also provides an unbiased and rapid stereoscopic approach for evaluation of targeted neuroprotection interventions. The protocol was validated by quantifying and comparing the absolute number of principal neurons and GABAergic interneurons within the rat hippocampus subfields with widely acceptable literature counts in age-matched rats.

## Materials and Equipment

### Animals

Young adult male rats (250–350 g) of the Sprague-Dawley strain (*n* = 11) were used in the study. The rats were housed in an environmentally controlled animal facility with a 12 h light/dark cycle. After 2 weeks of acclimatization to the vivarium, animals were utilized for the procedures. Animals were cared for in strict compliance with the guidelines outlined in the National Institutes of Health Guide for the Care and Use of Laboratory Animals. All animal procedures were performed according to a protocol approved by the university’s Institutional Animal Care and Use Committee.

### Stereology System

**Figure 1:**
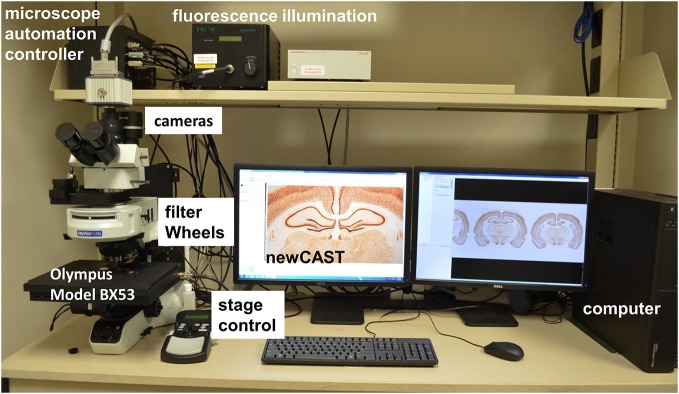
**A computer-driven stereology system.** Microscope automation controller manages stage movements (XYZ) of Olympus BX53 microscope, fluorescence filter and encoder for stereology newCAST program. For epi-fluorescence, the fluorescence illumination, filter wheels and specific CCD camera system (ORCA) are shown.

The stereology system consists of an Olympus BX53 microscope (Olympus, Tokyo, Japan) fixed with a DP73 cooled digital color camera (Model: DP73-1-51, Olympus, Tokyo, Japan) or ORCA-R2 digital CCD camera (Hamamatsu, Hamamatsu City, Japan) for immunofluorescence images (Figure 1). A motorized stage (Model: H101ANNI, Prior Scientific, Rockland, MA, USA) controlled by universal microscope automation controller with encoder (Model: 500-H31XYZEF, ProScan III, Prior Scientific, Rockland, MA, USA) and Proscan III joystick (Model: P-PS3J100, Prior Scientific, Rockland, MA, USA) makes the protocol and hardware more user-friendly. The BX53 microscope is fixed with a 1.25× objective (PLAPON1.25×, numerical aperture (NA) = 0.04, working distance (WD) = 5.1 mm, Olympus), 10× objective (UPLSAPO10X2, NA = 0.4, WD = 3.1 mm, Olympus), 20× objective (UPLSAPO20XNA = 0.75, WD = 0.65 mm, Olympus) and 60× oil immersion objective (UPLSAPO60XO, NA = 1.35, WD = 0.15 mm, Olympus). Olympus immersion oil type-F (IMMOIL-F30CC, Olympus) was used with the 60× oil immersion objective. Appropriate cleaning supplies should be readily available for use such as lens cleaning solution, lens paper, and 99% ethanol for cleaning the slides. The stereology software used in this protocol is newCAST (Version: VIS4.6.1.630, Visiopharm, Denmark). A calibration slide with a standard Visiopharm calibration grid embedded on the glass sheet in the center is included with the newCAST VIS software. The calibration slide is to be used for calibrating the center of the view and size of the field-of-view under a series of magnifying lenses in the microscope. The calibration step has to be done before any stereology procedure.

## Stepwise Procedures of Stereology

### Perfusion and Sectioning

Rats were anesthetized with ketamine-xylaxine mixture and transcardially perfused with 4% formaldehyde solution (Fisher Scientific) in sodium phosphate buffer (PB, pH 7.4). Following this, the rats were decapitated and the brain was carefully removed from the skull of each animal and post-fixed in 4% formaldehyde for 16 h at 4°C. The brain tissues were next treated with PB (for 24 h), 10, 20 and 30% sucrose solution in PB for 72 h each respectively and then rapidly frozen with O.C.T compound (Sakura Finetek, CA, USA) on dry ice (Rao et al., 2006; Kuruba et al., 2011). Serial sections (30 μm thick) were cut coronally through the rat forebrain containing the amygdala and the hippocampus, approximately from bregma −0.24 to −7.44 mm (Paxinos and Watson, 2007). The sections were collected serially in 24-well plates filled with PB. Every 20th section through the entire rat hippocampus (every 10th section for mice) was then selected from at least six animals. Sections were taken at 600 μm intervals for rat (300 μm for mice) and processed for neuronal nuclei antigen (NeuN, total neurons) and parvalbumin (PV, interneurons)-immunoreactivity. The first set of sections from one randomly selected starting section was processed for Nissl staining (all cells). The second series of sections were processed for NeuN immunohistochemistry to visualize all neurons within the brain. Additional series of sections were processed for PV immunohistochemistry, by which the PV+ interneurons in different subfields of the hippocampus were visualized. Post-sectioning measurements revealed minimal variability in slice thickness. However, sections showed significant thickness shrinkage along the *Z*-axis following NeuN immunostaining. The average thickness of the sections was reduced to 52–60% of the initial section thickness (*n* = 32). The difference in overall shrinkage of sections from different subjects was insignificant between groups.

### NeuN Immunohistochemistry

The procedure employed for NeuN immunohistochemistry was based on previous report in Rao et al. (2006) and Hattiangady et al. (2011). The sections were processed for NeuN-immunoreactivity with the specific monoclonal mouse anti-NeuN antibodies (Cat# MAB377, Millipore, Temecula, CA, USA). The sections were rinsed in 0.1M phosphate buffered saline (PBS, Sigma) and incubated with 20% methanol and 3% hydrogen peroxide for 20 min and washed thrice with PBS. After inactivating the endogenous peroxidase activity with hydrogen peroxidase, they were washed in 0.1 M PBS solution. Next, sections were treated with 10% normal horse serum and 0.1% Triton X for 30 min to block non-specific binding of antibody. Furthermore, sections were incubated overnight in PBS containing the normal horse serum, Triton X-100 and the specific NeuN antibody (1:1000, Millipore) at 4°C. Subsequently, the immunoreaction product was visualized according to the avidin-biotin complex (ABC) method (Hattiangady et al., 2011) using the Vectastain elite ABC kit (Cat#: PK-6100, Vector Lab, Burlingame, CA, USA) and 3^′^,3^′^-diaminobenzidine as a chromogen (Cat#: SK-4100, Vector Lab, Burlingame, CA, USA). Following thorough washes in distilled water, all sections were mounted on gelatin-coated slides, air dried, dehydrated in ethanol, cleared in xylene, and cover slipped (Cat#: 48393-059, VWR, Radnor, PA, USA) with DPX (Sigma).

### PV Immunohistochemistry

The procedure employed for PV immunohistochemistry was outlined in previous report in Kuruba et al. (2011). Briefly, the sections were first processed for etching, which involved immersion of sections in PBS solution containing 20% methanol and 3% hydrogen peroxide for 20 min. Sections were next rinsed three times in PBS, treated with 10% normal horse serum in PBS containing 0.1% Triton X-100 and incubated overnight in a mouse anti-parvalbumin (PV, Cat# P3088; Sigma-Aldrich, St. Louis, MO, USA) antibody solution (1:2000 in PBS). Subsequently, the sections were bathed thrice in PBS, treated with the biotinylated anti-mouse IgG solution (Cat#: BA-9200, 1:200, Vector) for 60 min, washed three times in PBS, and treated with the ABC reagent (Vector) for 60-min, as per manufacturer’s instructions. The peroxidase reaction was developed using diaminobenzidine as a chromogen (Vector Labs). The sections were placed on gelatin coated slides, dried overnight, dehydrated, cleared, and cover slipped.

### Stereology Imaging and Drawing

Super images of the entire slide containing hippocampal sections (three slices in each slide) were acquired by stereology newCAST software (Visiopharm, Denmark) with a 1.25× objective on the Olympus BX53 microscope. During super image capturing procedure, many individual field images are stitched together as a single super (large) image (Table 1; Figure 3). The Olympus BX53 microscope has an open field of view and motorized stage making the equipment user-friendly (Figure 1).

**Table 1:** **Step process for stereology**.

1. Turn on Microscope
2. Open visiopharm VIS software and click NewCAST tab
3. Open up super image software by clicking on the “super lens navigator”

**A. Capturing Super Image**
1. Load slide and select and focus the 1.25× objective
2. Click “Capture Super Images” from the drop down list, select “capture rectangle”
3. Under capture rectangle unclick “Define new Rectangle on Next Capture”
4. Navigate to the top left corner of the slide, click set under “Top/Left Corner (mm)” and repeat for the “Bottom/Right Corner (mm)”
5. Click “Capture Super Images” no blemishes
6. Use “Flat Field Correction” and find a region on the slide that has
7. Click Finish

**Figure 2:**
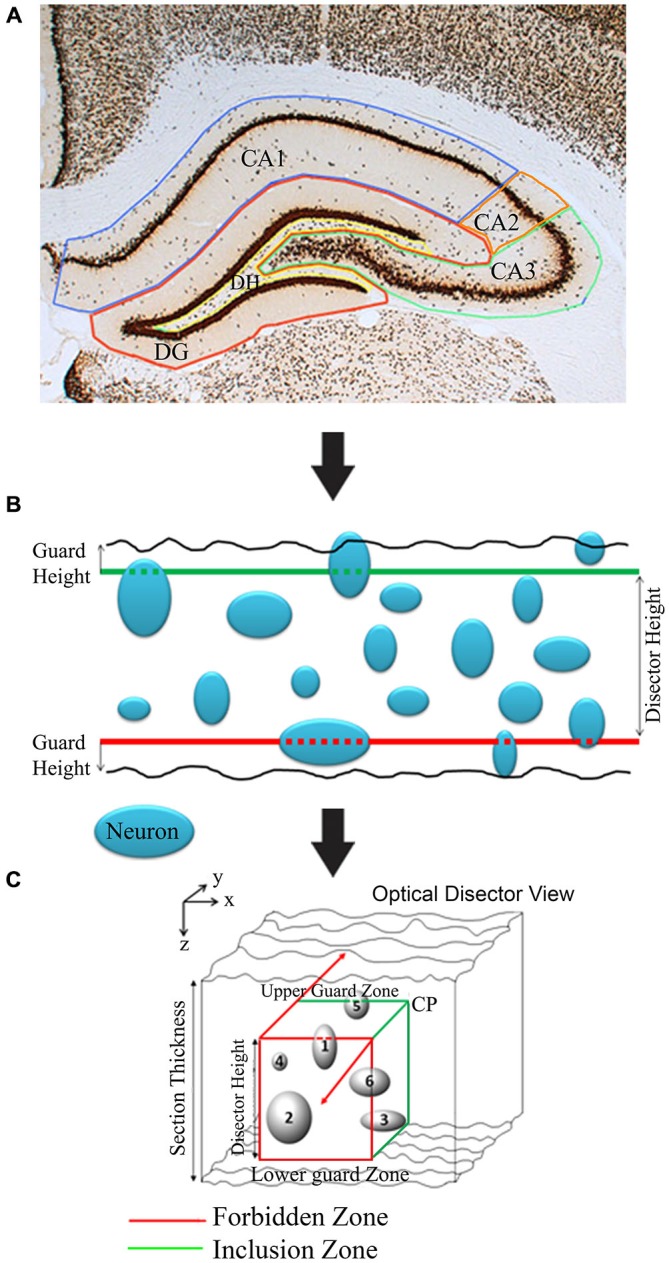
**Diagrammatic illustration of stereological analysis of neuron counting utilizing Olympus newCAST stereological system. (A)** Brain structure (e.g., CA1, CA2, CA3, DG and DH in hippocampus and also see Figure 6) markings for stereology. **(B)** Illustration of neuronal counts in 2D frames. **(C)** Illustration of optical dissector 3D-view for stereological estimations. Any neuron(s) (cells 1 and 6) touching red line/zone is excluded from count. CP: Corner point.

**B. Drawing (Figures 2, 6)**
1. Save the super image using the floppy disk icon
2. Click on the “Layer Drawing Mode” Button
3. Delineate the regions of interest (ROIs): CA1, CA2, CA3, DG, DH
- The ROI outline is finished with a double-click

**C. ROI Volume (Figure 4)**
1. Select and focus the 10× objective
2. Click on the “Points Probe”
3. Turn the points on and refer to Table 2 to set the point dimensions for the ROI
4. Click on “New Data Sheet”
5. Click “Meander Sampling”
6. Choose the ROI necessary for the count
- unselect “Random Orientation”
7. Being the count by clicking on every point that is within the boundaries of the ROI
8. Click “Finish” when the count is completed
9. Repeat steps until every ROI is accounted for

**D. Volume Calculation (Figure 4)**
1. Select the file from the list of counted data
2. Click on “Perform Calculations on Database Data”, click import
3. Under “Type and Formula” choose “Stereology”
4. Choose “Cavalieri Estimator (V)”
-under “point grid counts” select “Mark 1”, under “Section Distance” type in “30 μm”

**E. Cell Density**
1. Select and focus the 60× oil immersion objective
2. Active the “Counting Frame”
3. Click on “New Data Sheet”
4. Click on “Meander Sampling” and choose the ROI necessary for the count
5. Select “Random Orientation”
6. Open the Z navigator
7. Select “Corner Point (CP)” in the “Count Tool” box, count the top right corner point of the frame if it is within the ROI
8. If a neuron is present inside the sampling frame, select “Mark 1 (or individual marker for CA1, CA2, CA3, DG or DH)” in the “Count Tool” box
9. Click Finish and save data
10. Repeat until all ROI have been accounted for

**F. Cell Density Calculation**
1. Select file from the list of counted data
2. Click on “Perform Calculations on Database Data”, click import
3. Under “Type and Formula” choose “Stereology”
4. Choose “Optical Fractionator”
- under “Counts” select “Mark 1”, for “bsf”, “ssf” and “asf” type in the values “1.0”, “0.05” and “0.5”
5. For the second cell density calculation, repeat steps 1–3, then choose “Optical Disector”
- under “Counts [Q^−^]” select “Mark 1”, under “Counting Frame Corner Points” select “CP”, type in 30 μm for “Block Advance”
6. Repeat for all ROI


**Figure 3:**
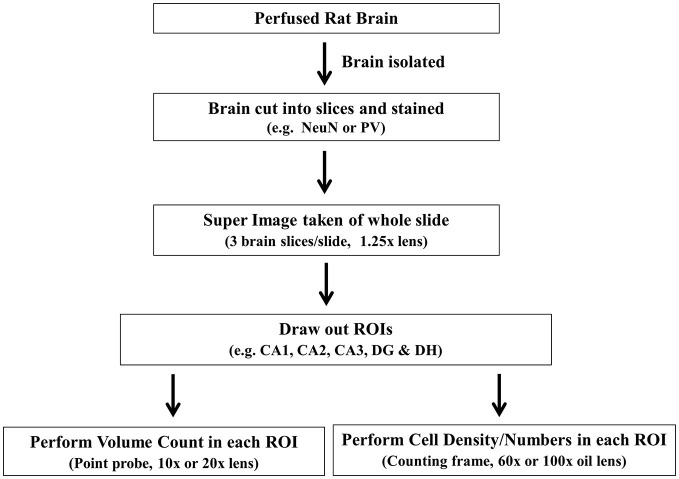
**Flow chart shows the general outline of stereology protocol.** ROI, region of interest.

**Figure 4:**
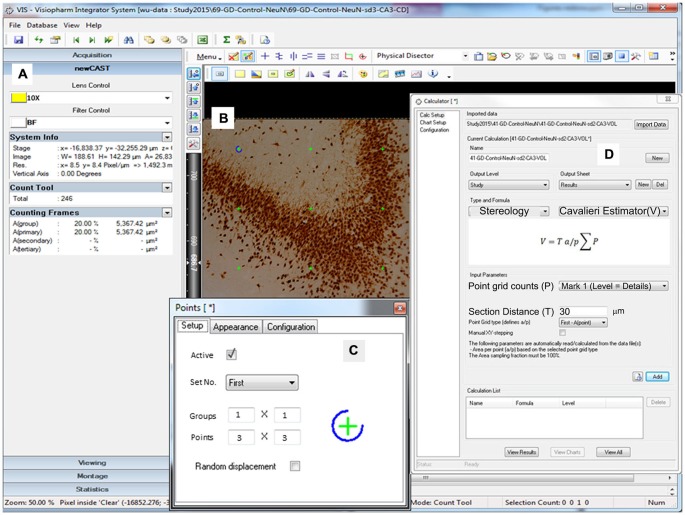
**Representative screen shot illustrations.** Screen depicting the volume crosshairs (green color, **B**) in a 3 × 3 pattern (**C**, Points: 3 × 3) through the 10× objective lens (**A**, Lens Control). **(D)** Window illustrating the calculation screen for the Cavalieri estimator of volume (V) along with the formula utilized.

**Figure 5:**
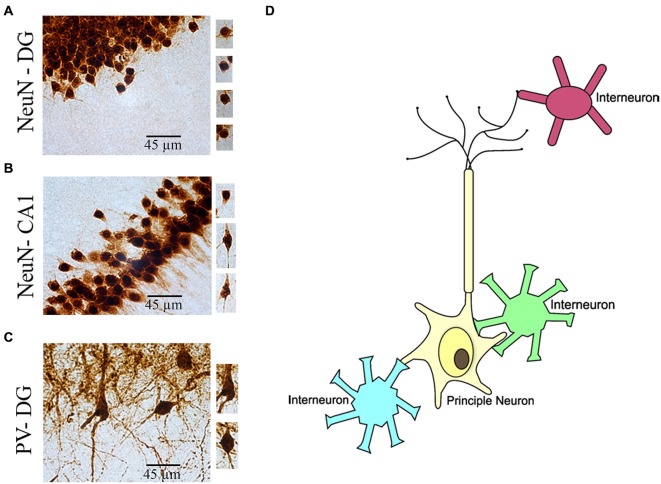
**Illustration of morphological identification of principal neurons and interneurons in the brain. (A)** NeuN(+) granule cells in the hippocampus dentate gyrus (DG) region. **(B)** NeuN(+) pyramidal cells in the hippocampus CA1 region. **(C)** PV(+) interneurons in the hippocampus DG region. **(D)** Cartoon illustration of link between principle neuron and interneurons in the brain.

**Figure 6:**
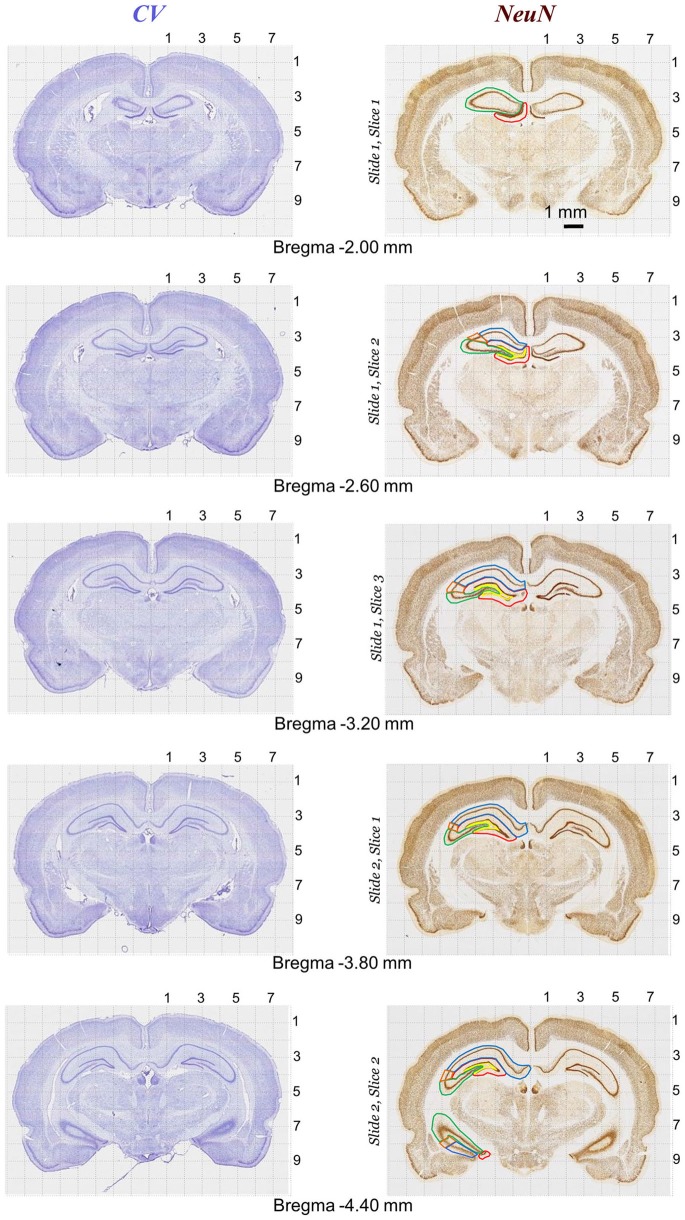

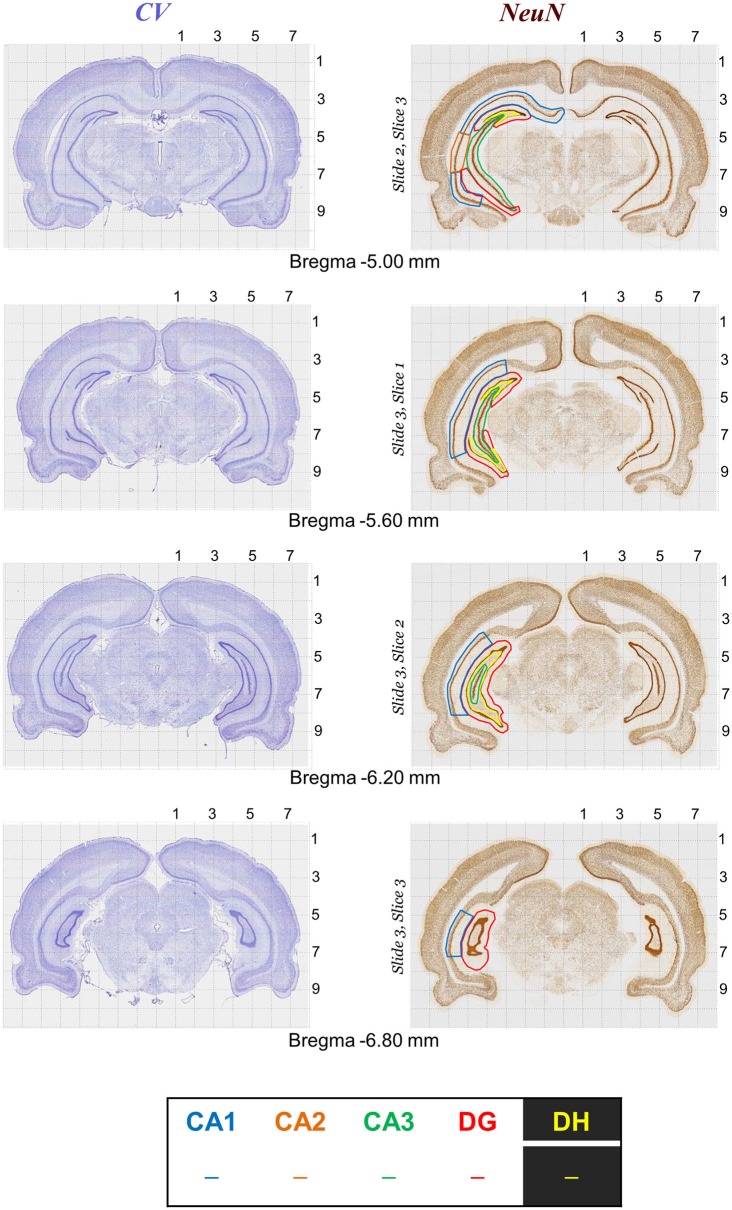
**Rat brain atlas illustration of quantitative neurostereology protocol in sections stained with Nissl (left panels) and NeuN staining (right panels).** Brain structure (e.g., CA1, CA2, CA3, DG and DH in hippocampus) markings for stereology.

To begin cell counting, each section of the hippocampus was delineated into five contours: (1) dentate gyrus (DG); (2) dentate hilus (DH); (3) Cornu Ammonis region I (CA1); (4) Cornu Ammonis region II (CA2); and (5) Cornu Ammonis region III (CA3; Figures 2–6). This was done according to the Paxinos and Watson rat brain atlas (Paxinos and Watson, 2007). This is accomplished using the tracing function of the newCAST software. Neuronal density in the different regions of the hippocampus appeared mostly symmetrical between the two hemispheres of the brain, thus quantification was performed only on one side, chosen at random. A brief step-by-step process for cell counting is given in Table 1.

### Tissue Volume

The volume of any specific ROI can be found by utilizing the 10× objective lens in the Olympus BX53 microscope. Cavalieri theorized that two solids will have the same volume when they are the same height and when their cross sections are taken in a parallel manner respective to their bases along with the same distance respective from the bases (West, 2012a). Therefore, we are able to calculate the overall volume of a delineated section by looking at a fraction of its slices that lie in equal interval; thus saving an exponential amount of time. However, in order to calculate the volume without bias, we combined Cavalieri’s theory with a process known as vertical sectioning (Baddeley et al., 1986) in order to produce more effective results. The Visiopharm’s imaging software allows users to combine these protocols in order to quickly and efficiently produce the volume of a ROI.

To produce an accurate volume estimation, at least 200 points were required to be counted in each ROI. For example, for 10 sections of tissue, on average, 20 crosshairs overlaying the ROI in each section were counted (West, 2012a, 2013). Initially, the points probe was set to one group of 3 × 3 crosshairs that will appear as a guide on the monitor (Figure 4; Table 2). The only ROIs that had a differentiating number of crosshairs were the DH and CA2 (5 × 5) due to their miniscule size in comparison with the larger regions of the hippocampus. Only crosshairs that appear within the ROI were tallied. The others were disregarded in order to allow the program to correctly determine the overall volume. The entire delineated section of the hippocampus was analyzed at 100% sampling and volume was not taken randomly (Figure 4). The section distance was set for 30 μm as our cutting thickness for volume estimation.

**Table 2:** **Counting references for stereological analysis of neuronal tissue volume and cell density for NeuN and parvalbumin immunostaining in rat**.

	Volume counting (Points, Non-random), 10× lens			Optical disector (Counting Frame, Random), 60× oil lens
	Sampling (%)	Group	Points	Meander sampling fraction (%)
CA1	100	1 × 1	3 × 3	5
CA2	100	1 × 1	5 × 5	10
CA3	100	1 × 1	3 × 3	5
DG	100	1 × 1	3 × 3	5
DH	100	1 × 1	5 × 5	10

*Group and Points for total tissue volume and percentage of meander sampling fraction for optical disector (cell number) are subject to change based upon region of interest (ROI) and staining type.

### Optical Fractionator and Sampling Scheme Method

The optical fractionator combines the optical disector, a 3D probe used for counting neurons, and the fractionator, a systematic uniform sampling scheme of a known fraction of the region being analyzed. To yield an unbiased total neuronal count in a brain region, all sampling should be accomplished systematically and randomly. The fractionator concludes that a quantity known for a small fraction of a structure can be used to determine the quantity of the entire structure (Gundersen, 1977; Charleston, 2001; Dorph-Petersen and Lewis, 2011). The optical fractionator method samples a known fraction of the section thickness, under a known fraction of the area of the region containing the neurons under consideration, on a known fraction of the sections that pass through the hippocampus (Keuker et al., 2001; Boyce et al., 2010; Dorph-Petersen and Lewis, 2011).

The fractionator sampling scheme assumes that each portion of the hippocampal brain structure has equal odds of being sampled into frames. In addition, all positions in the plane of each section have an equal probability of being sampled with probes (Walker et al., 2002; West, 2012a,b). The sections were randomly selected from the brain region based on a predetermined, uniform interval. The first section’s position was randomly chosen within the interval, and the sections for the region thereafter are established once the first random position has been determined. The systematic random sampling method was extended throughout each sampling process involved in the stereoscopic approach—volume, cell density and neuronal count.

Design-based sampling, a procedure aimed at ascertaining that all particles in the sampling space have the same chance of being sampled, was adopted without making any preliminary assumptions about the morphology of the tissue/organ under analysis (Geuna and Herrera-Rincon, 2015). To obtain neuronal counts in the DG, CA1, CA2, CA3 and DH of each animal, NeuN(+) cells were counted from randomly selected frames, accounting for 5–10% of total region area. The frames were placed superficially over each of these cell layers in every twentieth section throughout the hippocampus. The percentage of area was increased to 10% when determining the number of neurons in the CA2 and DH to ensure a large enough sample was being analyzed (Boyce et al., 2010; Dorph-Petersen and Lewis, 2011). All NeuN(+) cells present in the optical disector frames (20% of field of view size) at 60× oil objective were counted in each of the regions of the hippocampus. The absolute cell number counts and densities were calculated using the optical fractionator component of the Visiopharm software. The sampling scheme chosen ensured that the sample concentration remained constant for each section. Thus, counting 5% of the DG, CA1 and CA3 or 10% of the CA2 and DH, effectively guaranteed every NeuN(+) neuron within the hippocampal regions had equal odds of being selected and counted. The optical fractionator is combined with the disector frame to find the total number of neurons (*N*) in a particular brain region. The number of neurons (*N*) is estimated as:

(1) $$\text{N = }\sum {\text{Q}}^{-}\cdot (\text{1/bsf})\cdot (\text{1/asf})\cdot (\text{1/hsf})$$

(2) $${\text{hsf = h/tQ}}^{\text{-}}$$

(3) $${\text{tQ}}^{\text{-}}=\sum_{\text{i}}\left({\text{t}}_{i}\cdot {\text{q}}_{i}\right)/\sum_{\text{i}}\left({\text{q}}_{i}\right)$$

In which (∑Q^−^) is the total neuron count in the brain region acquired with the optical dissector; *bsf* is the block sampling fraction; *ssf* is the section sampling fraction; *asf* is the area sampling fraction; *hsf* is height sampling fraction; *h* is disector height; tQ^−^ is the number-weighted mean section thickness; t_*i*_ is the measured section height at position *i* and q_*i*_ is the number of counts at position *i*. Every 20th section (10th section for mice) is collected in a series of sections for analysis (ssf) in the rats. The counting procedure is conducted within one hemisphere only (asf), randomly selecting the left or right hemisphere for analysis. Hence, when calculating the cell density or cell number (*N*), the settings for bsf, ssf, asf were fixed to 1.0, 0.05 (1/20th), and 0.5 (one side of hemisphere).

### The Optical Disector

The optical disector consists of an unbiased counting frame that is moved through a relatively thick portion of tissue by focusing the microscope. Neurons were counted as they came into focus within the frame. The disector frame consists of two in-bound green lines (inclusion lines) and two out-of-bounds red lines (exclusion lines; Figure 2C). Anything touching those red lines is not counted; the opposite is true for the green lines and the area within the cubic of disector height. Neurons that appeared to have a darkly stained nucleus are selected for calculation by marking the top of the nucleolus when it is focused. Counting the nuclei ensured that over counting did not occur, since there is only one nucleus for each neuronal cell. If a cell is not completely inside the disector frame, but is also not touching the out-of-bounds lines, it is still counted. The areas directly above the optical disector and directly below were considered to be guard zones that form another forbidden plane. These guard zones ensured that defects in the tissue such as cuts and pits were not counted in the disector volume associated with the frame (Figure 2).

The optical disector creates a cube of volume within a delineated portion of tissue, allowing for 3D counting on stacked 2D images. Counting with the disector technique eliminates potential bias as it involves direct counting of objects within a defined volume structure with random sampling. The tissue preparation allows the neurons to be easily identified, providing a definitive, dark nucleus staining with a light background. The optical disector also allows for all objects, regardless of shape, size and orientation, to have equal opportunities of being associated with the volume of the tissue within optical disector frame (West, 2012a; Geuna and Herrera-Rincon, 2015).

The newCAST software combines the optical fractionator sampling scheme with the optical disector so that the calculations can be performed at the same time. Therefore, the same sampling fractions and settings, as well as the 60× oil immersion lens were used as described in the optical fractionator section. As the sections progress throughout the overall region, two components are accounted for. If the upper right corner of the counting frame lies within the delineated section, a corner point (CP) is manually tallied for each time this occurs. Second, every intact neuron present within the constraints of the counting frame is marked for calculation. By utilizing the fine focus knob of the microscope, the optical disector found the volume of each frame that contains at least one intact neuron. The optical disector is moved through the *Z*-axis of the frame, starting at 0 μm where the field of top view (top surface) is marginally blurred, and ending when the field of view is blurred in the opposite direction. This process finds both the highest and lowest vertical guard heights for the optical disector, ensuring that the neuron is completely contained within the cube of volume created by the optical disector. Our results show that the thickness of the tissue averages between 52–60% of original thickness after shrinkage. The disector height is selected when 90% of cells in the field of the slice will be counted in the optical disector cubic (*n* = 32, Figure 2; Dorph-Petersen et al., 2001; West, 2012a). The combined design of fractionator and optical disector is not sensitive to shrinkage as the number of cells within a given fraction is not affected by shrinkage [formula equations (2) and (3)].

### Data Analysis

Data from brain sections from a group of 11 rats are expressed as the mean ± standard error of the mean (SEM). Comparison of mean values between groups was made with one-way analysis of variance, followed by unpaired two-tailed Student’s *t*-test. In all statistical tests, the criterion for statistical significance was *p* < 0.05.

### Stereological Parameters for Mice

Even though rats have been widely used for studying neuronal diseases, mice are used more frequently for studies involving genetic modeling, neuronal plasticity, and neurodegeneration. This stereology protocol can also be used to estimate the total number of neurons within a mouse brain, with a few changes in parameters. The main regions of the hippocampus will still be drawn out according to an atlas of a mouse brain. Volume will be taken with the 10× objective; however the number of crosshairs on the screen will be changed to 6 × 6 for the DG, CA1, and CA3 and 11 × 11 for the CA2 and DH. The mouse brain is considerably smaller than the rat brain, so increasing the number of crosshairs for the volume count will ensure that the volume will be more accurate. Lastly, the cell density will be performed in the same way as the protocol for the rat brain, but the percentages will be increased to 10% for the DG, CA1, and CA3, and 15% total volume area for the CA2 and DH.

## Anticipated Results and Discussion

### Neurostereology Protocol Optimization

We optimized a stereology protocol to suit targeted neuron counts within the rat hippocampus (Table 2; Figure 3). Before starting any slide, a quality check was done in every section based on our standard expectations for general brain architecture and immunostaining pattern. If there was any deviation from that expectation, such as staining issues, the concerns were resolved by re-staining the sample in a new set of sections. After successful verification of the sections and software parameters, the principle and interneuron cell counts were performed using various in-built features of the program (Figure 4). Principle cells and interneurons were identified based on their typical morphology and their anatomical context (Figure 5). Our optimized protocol allowed an unbiased quantification of total neurons in the hippocampus subfields in approximately 3 h, with the possibility of the completion of up to three animals in a single day. The protocol was utilized for quick quantification of NeuN(+) total neurons and PV(+) interneurons in the hippocampus subfields CA1, CA2, CA3, DG and DH (Figure 6).

### Quantification of Principal Neurons, Interneurons and Total Neurons in the Hippocampus Subfields

**Figure 7:**
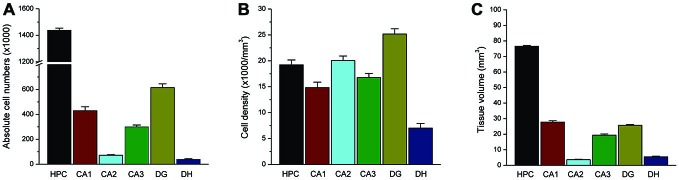
**Total NeuN(+) neuron counts within the rat hippocampus.** Bar graphs shows the total absolute number **(A)**, cell density **(B)** and subfield volume **(C)** of NeuN(+) principal neurons within the hippocampus subregion. Data represent the mean SEM (N = 11 rats per group).

**Figure 8:**
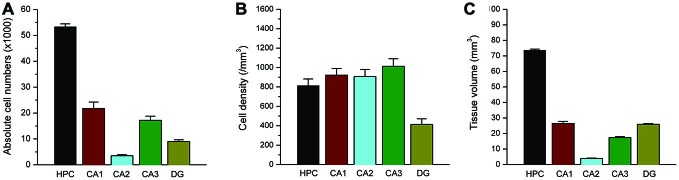
**Total PV(+) interneuron counts within the rat hippocampus.** Bar graphs shows the total absolute number **(A)**, cell density **(B)** and subfield volume **(C)** of parvalbumin (+) GABAergic interneurons within the hippocampus subregions. Data represent the mean SEM (N = 10 rats per group).

To validate the stereology protocol, the absolute number of total cells and interneurons were separately quantified in each hippocampus subfield in rat brain sections stained with NeuN or PV. The program was set for calculation of the cell density, using the 60× oil immersion lens, and tissue volume, using the 10× lens in a protocol considered as 3D counting. In the normal control rat, our NeuN(+) counting estimated a total of 1.5 million total neurons in the whole hippocampus in normal adult male rats (Figure 7). Subfield-specific distribution of PV(+) neurons in various subfields within the hippocampus was counted separately. Our PV(+) counts revealed a total of 55 thousand interneurons in the whole hippocampus in adult male rats (Figure 8). These numbers represent absolute total cells and are highly consistent with the literature reports of total neurons in the hippocampus in age-matched rats (West et al., 1991). Post-seizure rats can be expected to demonstrate strikingly reduced numbers of principal neurons in the DH along with the CA1 and CA3 pyramidal cell layers (Rao et al., 2006). Our protocol showed a similar pattern of neuronal loss following persistent seizure activity (Kuruba et al., 2014; Wu et al., 2014). Taken together, this protocol enabled us to accurately make medium-throughput counting of total neurons and interneurons in the brain as consistent with previous reports of total neurons in the hippocampus in age-matched rats (Pakkenberg and Gundersen, 1988; West et al., 1991; Rao et al., 2006).

### Discussion of Main Protocol

The present study shows successful optimization of a neurostereology technique for absolute quantification of specific types of neurons and interneurons in the brain sections. This stereology protocol allows sensitive, medium-throughput counting of total neurons in any brain region, consistent with comparable methods utilized for estimating neuronal injury or neurodegeneration. Depending on the specific neuroprotection intervention utilized, our protocol can provide accurate results for any brain section. The computer-driven system utilized in this study is similar to other leading systems, such as the StereoInvestigator System, that are commonly utilized in other neurodegenerative research labs (Rao et al., 2006). With both systems, important quantitative descriptions of the geometry of 3D structures can be completed from measurements made on stacked 2D images. This is accomplished using formulas that take into account pertinent structural frameworks that are lost during the formation of brain sections; thus providing an elegant quantitative tool for studies of neuroprotection or neurodegeneration in a variety of neurological and brain injury models. Our protocol is consistent with the previous reports on using stereological method counting a variety of neuron types including dorsal root ganglion cells after nerve injury (Sorensen et al., 2003), total neurons and glial number in the geniculate nucleus (Selemon and Begovic, 2007).

The present study has allowed us to describe different techniques used within our stereology protocol—Cavalieri’s estimation of volume, the optical fractionator sampling scheme and the optical disector for cell density and cell number. In order to obtain an accurate neuronal count, the cell density estimate has to be multiplied by the volume of the reference space (Royet, 1991)—each section of the hippocampus. The Cavalieri estimation of volume was used as the reference for the tissue volume. The densities of principal neurons and interneurons in the hippocampus (Figures 7, 8) are consistent with previous reports on absolute total neurons in the hippocampus in age-matched rats (Pakkenberg and Gundersen, 1988; West et al., 1991; Rao et al., 2006). However, some variation in the absolute numbers are apparent because of differences in protocol efficiency, strain and/or antibodies used for immunostaining (Aika et al., 1994; Nomura et al., 1997).

### Advantages of the Method

One of the advantages of the current method is that it combines several elements of similar stereological methods to create a more efficient process. The use of the fractionator greatly improves efficiency. It allows one to estimate the number of neurons in a given section of tissue without knowing the exact section thickness, volume of the reference section, area of the sampling frame and the magnification (Royet, 1991), thus removing a margin for error due to tissue deformation and tissue shrinkage during the fixation and embedding stages. Although errors in neuronal count may still occur from the subjective detection of neurons within the disector frame, the same biases would occur if the optical disector and fractionator methods were not being implemented in the process. The extent of this error, however, can be reduced by ensuring that adequate staining for neuron identification is used. Thus, our method of stereology protocol is reasonably fast and efficient; the calculations for section volume and cell density can be obtained in approximately 3 h per brain.

In this study, we used the normal rat hippocampus as a model region for the stereology protocol optimization. Nevertheless, this method can be easily adapted for any neurodegenerative disease in mouse or human models, as all neurodegenerative disorders share the similar feature of neuronal loss. Only NeuN(+) neurons and PV(+) interneurons were stained for and used in this protocol. However, this method can be used to count any number of items within a given region of tissue. Stereology has been used in a variety of different studies in the past, including estimating the length and size of myelin fibers in the white matter of a human brain (Tang and Nyengaard, 1997; Schettino and Lauer, 2013). Although we have not made attempts to extend our protocol for such measurement, it can be adjusted for quantification of the length and size of any cell or neuron in the brain.

Modern design-based stereology has been an important and efficient tool in brain research. The computer-aided optical dissector/fractionator system has been developed by many groups in the last two decades. Immunofluorescence has become a common technique in recent years and can also be analyzed using this protocol. In order to adjust this protocol to count fluorescent stains, the compound microscope would need to have the correct external devices, namely a fluorescence light illumination system (Figure 1). The nature of the fluorescent staining dictates that the compound microscope being used should also have a manually or automatically operable shutter system to control the amount of exposure to light. A procedure called microimager captures many 2D images of slices at once at different positions of the *Z*-axis and stacks the 2D images to create a 3D experience. The stacked 2D images can later be analyzed offline. The microimager reduces fluorescence light exposure to the slices and has the ability to switch between different filters, especially in the multiphoton situation.

Our optimized protocol, when combined with the boundless spectrum of usage within the field of stereology, has incredible applications within experimental neuroscience. This protocol may be utilized with many different forms of staining from crystal violet to antigen staining and provides an easy to follow model for users of all backgrounds. Even though this stereology protocol is currently used with a focus in neuroscience, our procedure holds considerable potential for immunology, pathological research, diagnosis and the clinical field (Gundersen et al., 1988; Jones et al., 2013; Cruz-Orive et al., 2014).

### Potential Problem Areas and Solutions

One of the most difficult challenges of stereology is defining the top and bottom positions of the slice under the microscope (Geuna and Herrera-Rincon, 2015). This is an issue especially when using fluorescence. Training for this step can be rigorous in order to be confident that correct results are being collected; however, this challenge can be overcome by placing markers in the top and bottom of the slices. Visiopharm Integrator System newCAST software also has tools that can be used in a variety of imaging procedures such as confocal microscopy for comparisons between subjects.

There are important issues with differential quantification of principal neurons and interneurons. NeuN immunostaining is widely used to label all neurons, both principal cells and interneurons. NeuN(+) cells provide an estimate of total neurons in the brain region. There are some claims that a few neuronal cell types are not recognized by the NeuN antibody; however, the vast majority of neurons are strongly NeuN(+) as they mature. Regarding interneuron counts, PV(+) neurons are one sub-class of hippocampal interneurons. These neurons represent up to 40% of the entire hippocampal GABAergic interneuron population. Therefore, additional interneuron markers, such as somatostatin, neuropeptide *Y* and cholecystokinin, may be helpful for absolute total interneuron counting.

Additional technical issues are outlined below: (1) Different batches of the same staining reagents (especially immunofluorescence reagents) might yield inconsistent results. Test the reagents before use for each new lot of reagent purchased. (2) During the immunohistochemistry procedure, dehydrating slices in the same situation and length to keep tissue shrinkage in the comparable range can also be difficult. (3) Tissue structure needs to be identifiable, results are more accurate when 100% of the tissue is available for analysis. Occasionally sections are torn or lost during processing and those animals should be discarded. (4) The measurement of dissector/tissue section thickness in each ROI along the *Z*-axis can be manipulated by many factors related to the slice (e.g., irregularity in the section shrinkage) and investigator’s experience with the optics (e.g., thickness of coverslips and type of lens). Performing a careful calibration and pilot study when using a stereological method is useful for avoiding bias related to the practical application. (5) Unbiased stereology methods are tedious and require extensive training. Identifying the top and the bottom of the slices along the *Z*-axis is somewhat difficult to detect. Find the first position in the *Z*-axis where something comes into focus in the center of the counting frame. Then continue moving in one-direction (up), and when the field of view is completely out of focus, set the current position to 0 (top position). Move down the *Z*-axis until nothing is in focus at the opposite end of the slide and push “stop” on the *Z* navigator for the bottom position of the slide. The field of view should be unfocused at the top, focused in the middle, and unfocused at the bottom of the slide to get an accurate counting frame volume.

### Conclusion

In conclusion, these results suggest that the optimized neurostereology protocol is a powerful tool for studies of neuronal injury and neurodegeneration by absolute quantification of total neurons and interneurons in the brain. This unbiased stereology method allows relatively rapid counting of total neurons in any brain region in a group of animals, and thus provides a quantitative tool for pharmacological investigations of neuronal injury or neuroprotection evaluations in animal models of neurological and brain injury conditions.

## Conflict of Interest Statement

The authors declare that the research was conducted in the absence of any commercial or financial relationships that could be construed as a potential conflict of interest.

## Abbreviations

NeuN: neuronal nuclear antigen
PV: parvalbumin
ROI: region of interest
DG: dentate gyrus
DH: dentate hilus
3D: three dimensional
AD: Alzheimer’s disease
PD: Parkinson’s disease
HD: Huntington’s disease
ALS: amyotrophic lateral sclerosis.
